# The AVOCAT study: Bicalutamide monotherapy versus combined bicalutamide plus dutasteride therapy for patients with locally advanced or metastatic carcinoma of the prostate—a long-term follow-up comparison and quality of life analysis

**DOI:** 10.1186/s40064-016-2280-8

**Published:** 2016-05-17

**Authors:** Siebren Dijkstra, Wim P. J. Witjes, Erik P. M. Roos, Peter L. M. Vijverberg, Arno D. H. Geboers, Jos L. Bruins, Geert A. H. J. Smits, Henk Vergunst, Peter F. A. Mulders

**Affiliations:** Department of Urology, Radboud University Medical Center, Geert Grooteplein Zuid 10, P.O. Box 9101, 6500 HB Nijmegen, The Netherlands; Department of Urology, Antonius Hospital, Bolswarderbaan 1, P.O. Box 20.000, 8600 BA Sneek, The Netherlands; Department of Urology, St. Antonius Hospital, Koekoekslaan 1, P.O. Box 2500, 3435 CM Nieuwegein, The Netherlands; Department of Urology, Slingeland Hospital, Kruisbergseweg 25, P.O. Box 169, 7000 AD Doetinchem, The Netherlands; Department of Urology, Koningin Beatrix Hospital, Beatrixpark 1, P.O. Box 9005, 7100 GG Winterswijk, The Netherlands; Department of Urology, Rijnstate Hospital, Wagnerlaan 55, P.O. Box 9555, 6800 TA Arnhem, The Netherlands; Department of Urology, Canisius Wilhelmina Hospital, Weg Door Jonkerbos 100, P.O. Box 9015, 6532 SZ Nijmegen, The Netherlands

**Keywords:** Bicalutamide, Dutasteride, Prostate cancer, PSA progression, Overall survival, 5α-Reductase inhibitor

## Abstract

**Purpose:**

Compare the efficacy and tolerability of dutasteride in combination with bicalutamide to bicalutamide monotherapy in the treatment of locally advanced and metastatic prostate cancer (PCa).

**Methods:**

One-hundred-fifty PCa patients with locally advanced or metastatic disease were prospectively enrolled and randomly assigned to receive either bicalutamide monotherapy 150 mg once daily (79 patients) or bicalutamide 150 mg plus dutasteride 0.5 mg once daily (71 patients). Treatment response was assessed by serum PSA level measurement, and standard procedures for diagnosis of clinical progression were used during follow-up. Patient-reported quality of life (QoL) was assessed using validated questionnaires (EORTC QLQ-C30 and QLQ-PR25).

**Results:**

At 3 years follow-up, PSA progression was found in 52 patients [65.8 %; 95 % confidence interval (CI) 55.4–76.3] in the monotherapy group compared to 38 patients (53.5 %; 95 % CI 41.9–65.1) in the combined therapy group (p = 0.134). At the time of analysis 37 men (46.8 %; 95 % CI 35.8–57.8) in the monotherapy group had died versus 30 men (42.3 %; 95 % CI 30.8–53.7) in the combined therapy group. Median survival time was 5.4 and 5.8 years, respectively (p = 0.694). There was no statistically significant difference in the presentation frequency of adverse events between groups (p = 0.683). QoL was good and comparable between the two groups.

**Conclusions:**

Both therapies were well tolerated with a good QoL. However, despite a trend toward higher efficacy of the combined therapy, progression-free survival and overall survival was not significantly different between the groups. Further research on this therapy should be performed.

## Background

Prostate cancer (PCa) is a major public health problem. With 29 % of all newly diagnosed cancers it represents the most common cancer among males, and with an estimated mortality of 9 % it is the second leading cause of cancer related death in this population (Siegel et al. 2012).

A rising serum PSA level is generally considered to be the earliest evidence of persistent or recurrent disease and between 20 and 40 % of men with clinically localised disease will eventually experience biochemical recurrence on long-term follow-up after primary treatment (Boorjian et al. 2012; Krygiel et al. 2005; Vickers et al. 2009). This is initially characterised by increasing serum PSA levels without radiographic evidence of a local recurrent tumour or distant metastases. PSA progression often precedes clinical progression and may signal the onset of this process (McLaren et al. 1998).

In case of disseminated disease, systemic treatment is highly indicated. Standard treatment for these patients is androgen deprivation therapy (ADT) using surgical or medical castration. The 5-year survival rate of patients subjected to ADT is 35 % (Studer et al. 2013). If considerable numbers of PCa patients receive prolonged ADT, decreased bone mineral density, increased risk for osteoporosis and skeletal fractures can ensue (Serpa Neto et al. 2010). Moreover, patients treated with ADT experience more erectile dysfunction, decreased sexual interest, activity and pleasure, and they report more frequent hot flushes in comparison to those not treated with ADT (van Andel and Kurth 2003).

Nonsteroidal antiandrogens, such as bicalutamide, bind to the androgen receptor and therefore the hypothalamic pituitary axis will not be blocked and testosterone levels are unaffected or slightly elevated, resulting in a clinical state without the adverse effects of ADT (Anderson 2003).

Furthermore, as a 5α-reductase inhibitor inhibits the intracellular conversion of testosterone to the more potent dihydrotestosterone, it is reasonable to consider that the combination of an antiandrogen and a 5α-reductase inhibitor should provide an effective form of maximal local androgen blockade (Wright et al. 1999).

And since testosterone concentration in plasma sustains, sexual function and quality of life (QoL) could be maintained (Iversen 2002). Using non-steroidal antiandrogens as a first line treatment in patients with localised or metastatic PCa might therefore delay ADT side-effects.

In this open-label prospective multicenter randomised clinical trial we evaluated the efficacy of bicalutamide monotherapy versus bicalutamide plus dutasteride combination therapy on overall survival and PSA progression in patients with locally advanced and metastatic PCa. Furthermore, the impact of both therapies on QoL was assessed.

## Patients and methods

In this multicenter study 150 patients were enrolled from 17 different sites throughout the Netherlands between 2006 and 2011. All patients had pathologically proven PCa and had indication for hormonal treatment, i.e. locally advanced or metastatic stage of disease, primary or progressive after treatment with curative intent. Additional eligibility criteria were a PSA level >10 ng/mL, a life expectancy of at least 12 months and no previous or concurrent chemotherapy or hormonal therapy specifically for the treatment of PCa. Approval was obtained from the Institutional Review Boards in accordance with the medical ethical requirements. After giving informed consent, patients were randomly assigned into one of the two treatment arms. One group received monotherapy bicalutamide 150 mg, orally once daily and the other group received combined therapy with bicalutamide 150 mg and dutasteride 0.5 mg, orally once daily.

Treatment response was monitored by serum PSA level measurement and biochemical progression was defined as an increase in PSA value ≥100 % of the nadir value on two separate determinations with an interval of at least 2 weeks. In case of PSA progression the decision was left to the discretion of the urologist, together with the patient, to opt for appropriate further treatment.

During follow-up patients underwent physical examination and assessment for adverse events. Patient-reported QoL was assessed using the validated European Organization for Research and Treatment of Cancer QoL core questionnaire (EORTC QLQ-C30) and the Prostate Cancer specific module (EORTC QLQ-PR25) (Lintz et al. 2003). These questionnaires were administered at screening, every three months thereafter and after 24 months annually, until discontinuation of treatment. Various pre-specified outcome parameters were extracted from these questionnaires, e.g. physical, emotional and social functioning, fatigue, pain, urinary/bowel symptoms and sexual activity.

### Statistical analysis

Sample size was set at 282 patients (141 patients in each treatment arm) and calculated to show a difference in proportions of patients with PSA progression of 30 % in the bicalutamide arm compared to 16 % in the combined treatment arm at 3 years follow-up (power 0.8, alpha 0.05). Anticipating a dropout rate of approximately 15 %, 324 patients were needed. The intended study duration was 3 years for recruitment and 3 years for follow-up. The study was discontinued after 4 years due to poor recruitment in the predetermined time frame for inclusion. Analysis was based on an intention-to-treat population. Statistical significance was assessed using the Pearson-Chi square test for proportions and the Wilcoxon–Mann–Whitney test for continuous variables. Overall and progression-free survival was calculated by the Kaplan–Meier method and comparison between the treatment groups was done by means of the log-rank test. Data from the QoL questionnaires were transformed linearly to obtain scores from 0 to 100 (Fayers et al. 2001). QoL comparison of the two groups was done using the independent samples *T* test. Statistical Package for the Social Science (SPSS) version 20.0.0.1 was used for analysis.

## Results

From September 2006 through August 2010 a total of 150 patients were eligible for participation in the study and enrolled for randomisation. 79 patients were assigned to the bicalutamide monotherapy arm and 71 patients received the bicalutamide and dutasteride regimen. Table 1 displays the baseline characteristics of all randomised patients.

**Table 1:** Baseline characteristics of eligible randomised patients

	Bicalutamide (n = 79) Median (range)/n (%)	Bicalutamide + dutasteride (n = 71) Median (range)/n (%)	P
Age, years	71 (54.2–86.1)	73 (54.9–90.7)	0.307^†^
Baseline PSA (ng/mL)	43.4 (11.6–15,000)	46.3 (11.3–660)	0.397^†^
*Clinical stage*			0.561*
T1	2 (2.5)	5 (7.0)	
T2	12 (15.2)	7 (9.9)	
T3	49 (62.0)	47 (66.2)	
T4	8 (10.1)	7 (9.9)	
Tx	8 (10.1)	5 (7.0)	
*Metastatic stage*			0.746*
M0	45 (57.0)	39 (54.9)	
M1	24 (30.4)	26 (36.6)	
Mx	10 (12.6)	6 (8.5)	
*Gleason score*			0.767*
≤6	16 (20.3)	12 (16.9)	
7	25 (31.6)	28 (39.4)	
8	13 (16.5)	12 (16.9)	
9	10 (12.7)	7 (9.9)	
10	2 (2.5)	0 (0)	
Missing	13 (16.5)	12 (16.9)	
*Primary treatment*			0.010*
Radical prostatectomy	6 (7.6)	3 (4.2)	
Radiotherapy treatment	12 (15.2)	27 (38.0)	
None	49 (62.0)	36 (50.7)	
Missing	12 (15.2)	5 (7.0)	

* Pearson Chi square test; ^†^Wilcoxon–Mann–Whitney test

At three years follow-up, PSA progression was found in 52 patients [65.8 %; 95 %confidence interval (CI) 55.4–76.3] in the monotherapy group compared to 38 patients (53.5 %; 95 % CI 41.9–65.1) in the combined therapy (CT) group (p = 0.134). This results in a difference between two independent proportions of 12.3 % (95 % CI −3.3−27.2) in favour of the CT group.

At three months of treatment 96.9 % (95 % CI 92.6–100) in the monotherapy group and 93.2 % (95 % CI 86.8–99.6) in the CT group had >50 % decrease in serum PSA levels. However, the mean nadir was significantly lower in the CT group compared to the monotherapy group [4.1 ng/mL (range <0.1–54.0) and 9.3 ng/mL (range <0.1–110.0); p < 0.001, respectively]. In the monotherapy group 11.5 % (95 % CI 3.5–19.5) reached a non-detectable PSA nadir during treatment compared to 39.3 % (95 % CI 26.5–52.1) in the CT group.

Median [interquartile range (IQR)] follow-up was 4.1 (2.0–5.5) years in the monotherapy group and 3.8 (2.0–5.4) years in the CT group. Sixty-nine (87.3 %; 95 % CI 80.0–94.7) patients discontinued treatment medication at these follow-up intervals in the monotherapy group compared to 55 (77.5 %; 95 % CI 67.7–87.2) in the CT group (p = 0.111). Median time to switch to further treatment was 1.6 (IQR 0.8–3.1) years versus 1.9 (IQR 0.9–3.9) years, respectively. The main reason for discontinuation of study medication was biochemical (PSA) progression (83.9 %; 95 % CI 77.4–90.3).

**Fig. 1:**
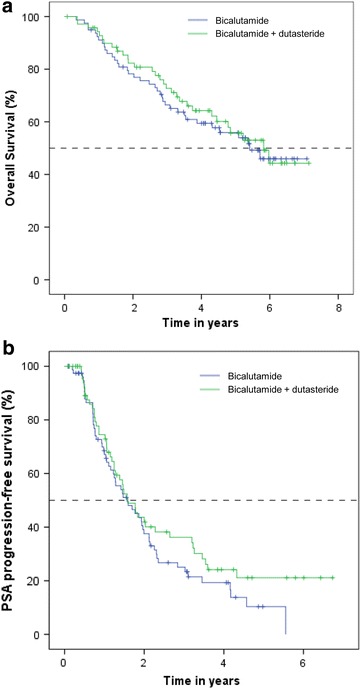
Kaplan-Meier estimates of overall survival (**a**) and biochemical progression-free survival (**b**) in the bicalutamide group and the bicalutamide plus dutasteride group

At the time of analysis, 67 of 150 patients (44.7 %; 95 % CI 36.7–52.6) died, 37 of 79 (46.8 %; 95 % CI 35.8–57.8) in the bicalutamide group and 30 of 71 (42.3 %; 95 % CI 30.8–53.7) in the CT group. No statistically significant difference was seen for overall survival between the two groups (log rank p = 0.694; Fig. 1a). There was a trend for better biochemical progression-free survival in the CT group, however, the difference was not statistically significant (p = 0.188; Fig. 1b).

### Adverse events

In total 108 adverse events were registered for 42 patients, 21 patients (60 events) in the monotherapy group versus 21 patients (48 events) in the CT group. Forty-one events (24 versus 17, respectively) were suspected to be related to the study medication. The most common adverse events were gynaecomastia (27.9 %) and nipple tenderness (18.6 %). Other minor adverse events were tiredness/fatigue, constipation, hot flushes and itch. There was no significant difference in the frequency of adverse events between the study groups (p = 0.683).

### Quality of life analysis

**Fig. 2:**
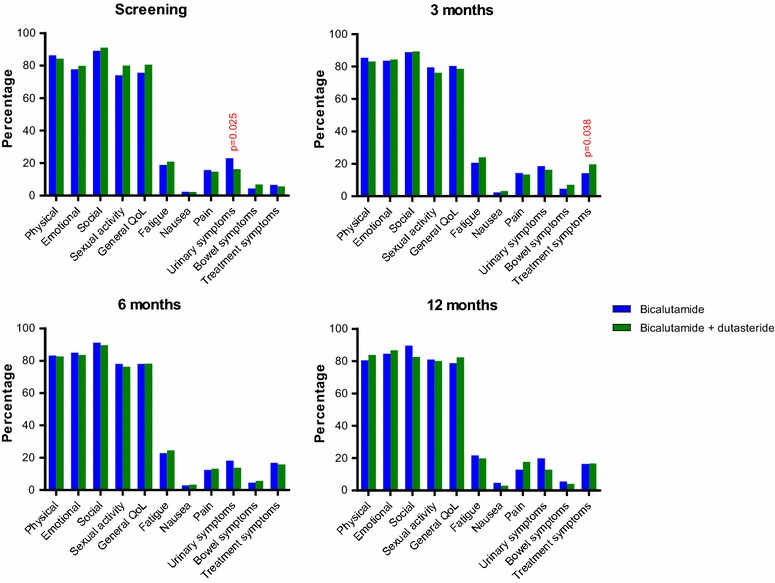
Quality of life outcome in both groups on five functional scales (physical, emotional, social, sexual functioning and general quality of life) and six symptom scales (nausea, pain, urinary, bowel and treatment related symptoms) at screening, 3, 6 and 12 months of treatment. A high score for general quality of life or a functional scale reflects a high QoL or a high level of functioning, whereas a high score for a symptoms scale implies a high level of symptoms

At baseline, data on QoL was available from 133 of 150 patients (89 %); 66 (84 %) in the monotherapy group and 67 (94 %) in the CT group. On the pre-specified outcome parameters no statistically significant differences were seen at screening between the two randomised treatment groups, except that the bicalutamide group reported more urinary symptoms (p = 0.025) (Fig. 2). After three and 6 months of treatment 62 (78 %) and 51 (65 %) QoL questionnaires were available in the monotherapy group and 62 (87 %) and 56 (79 %) in the CT group, respectively. At 3 months, patients in the CT group reported significantly more treatment related symptoms (hot flushes, painful nipples, weight gain/loss) compared to the monotherapy group (p = 0.038), however, this difference was not seen later and no other significant differences were found between the two treatment groups at any other timepoint (Fig. 2).

Functional QoL scales scored over 80 % in both groups, reflecting a high level of functioning on physical, emotional and social level. General QoL at baseline, 3, 6 and 12 months was maintained 76, 80, 78, 79 % in the bicalutamide group and 81, 78, 78, 82 % in the CT group, respectively. Sexual activity was scored 78 and 76 % at 6 months and 81 and 80 % at 12 months in the bicalutamide and CT group, respectively.

## Discussion

In this study we aimed to assess and compare the efficacy of bicalutamide monotherapy and bicalutamide plus dutasteride treatment in patients with locally advanced and metastatic PCa.

The number of patients to be recruited according to the sample size calculation was not reached and due to poor recruitment in the predetermined time frame for inclusion the study was aborted at the time of 150 enrolled patients. Nevertheless, the calculated difference of PSA progression between the groups at 3 years follow-up was 12.3 % (95 % CI −3.3–27.2) in favour of the CT group. This might be assigned to the added 5α-reductase inhibitor, however, due to the fact that the study was underpowered and the difference was not statistically significant, this assumption remains questionable.

Long-term follow-up studies demonstrated the benefit of bicalutamide on progression-free survival, whereas various other studies showed the efficacy of dutasteride in reducing the risk of incident PCa detection in patients at risk for PCa, reducing PCa progression in men with low-risk disease at active surveillance and reducing biochemical progression in patients with biochemical failure after radical therapy (Iversen et al. 2006, 2010; Andriole et al. 2010; Fleshner et al. 2012; Schroder et al. 2013).

Rationally it was reasonable to contemplate that adding a 5α-reductase inhibitor to the treatment with a pure antiandrogen such as bicalutamide should provide an effective form of maximal local androgen blockade. Monk et al. demonstrated the feasibility of combining flutamide and finasteride treatment and showed its efficacy regarding PSA response in a cohort consisting of 99 patients with a rising PSA after definitive local therapy (Monk et al. 2012). In a comparable study, Baňez et al. investigated the efficacy of the combination therapy flutamide plus finasteride compared to low-dose flutamide monotherapy in 56 men with biochemical recurrence after definitive therapy for PCa (Banez et al. 2009). However, after a median follow-up of 4.1 years they were not able to demonstrate a significant difference in biochemical progression-free survival between the two treatment arms. Nevertheless, on multivariate analysis, men on CT had a significantly lower risk of progression compared to men on monotherapy (Banez et al. 2009).

Since in these studies the lesser potent selective 5α-reductase inhibitor finasteride was used, we expected to find similar or even better results in our study. Yet, the fact that no significant benefit had been found in terms of biochemical progression-free survival or overall survival might be explained by the fact that our study was underpowered. Another explanation might be sought in the fact that our study consisted of patients with a high baseline PSA level (median 43.5 ng/mL), at least 33 % of patients was diagnosed with distant metastases and a majority had not undergone primary treatment with curative intent (64 %). This might indicate a more high risk patient cohort compared to the earlier mentioned study of Baňez et al.

In contrast to surgical castration and ADT, serum testosterone levels remain above castration levels in patients on antiandrogens and/or 5α-reductase inhibitor treatment, and thus sexual function and QoL will be better preserved. In this study we showed that patients in both groups had high levels of social functioning, sexual functioning and general QoL throughout the entire study. Our findings are confirmed by a study by Prezioso et al. This group showed an advantage of antiandrogen treatment compared to ADT, delaying ADT related side effects. Moreover, antiandrogen monotherapy was associated with a significantly better QoL on almost all functional scales compared to ADT (Prezioso et al. 2007). Since patients may require many years of treatment, tolerability and QoL are of increasingly importance when selecting the most appropriate hormonal therapy.

In our study, only a few treatment related adverse events were registered. Median time patients stayed on treatment medication before further treatment was initiated, was 1.8 years (22 months), which is comparable to the time that patients on ADT develop progressive disease. In a previous study by Tay et al., 86 % of patients remained responsive to subsequent LHRH agonists after earlier bicalutamide plus a 5α-reductase inhibitor treatment, indicating that this treatment as primary hormonal therapy for advanced PCa probably may not compromise the overall duration of the androgen-responsive disease (Tay et al. 2004). Since no data was available on further treatment, unfortunately we were not able to confirm this finding in our study.

Although a recent Cochrane review demonstrated the inferiority of anti-androgen monotherapy compared to ADT or surgical castration in overall survival and clinical progression, earlier mentioned studies on antiandrogen combination therapy showed promising results (Monk et al. 2012; Banez et al. 2009; Kunath et al. 2014). Moreover, side-effects of antiandrogen therapy might be more favourable compared to (medical or surgical) castration, however, a head-to-head comparison was not performed in this study. And despite a trend toward higher efficacy of the combined therapy was recognised, this study was not able to demonstrate a significant difference in efficacy between bicalutamide monotherapy and bicalutamide plus dutasteride combined therapy. Therefore, further research on this therapy should be performed.

## Abbreviations

PCa: prostate cancer
PSA: prostate-specific antigen
QoL: quality-of-life
ADT: androgen deprivation therapy
LHRH: luteinizing-hormone-releasing hormone
OS: overall survival
PFS: progression-free survival
